# Utilization of the gastrocnemius flap for post-traumatic knee reconstruction: a systematic review

**DOI:** 10.1007/s00590-024-03938-2

**Published:** 2024-04-18

**Authors:** Rohun Gupta, Joseph Weisberger, Isabel Herzog, Jacquelyn Roth, Edward S. Lee

**Affiliations:** 1 Oakland University William Beaumont School of Medicine Auburn Hills, Rochester, MI USA; 2 Department of Plastic Surgery, University Hospital, Newark, NJ USA; 3 Rutgers New Jersey Medical School, Newark, NJ USA

**Keywords:** Gastrocnemius flap, Post-traumatic knee reconstruction, Traumatic knee injuries, Post-traumatic knee pain, Soft tissue defects

## Abstract

**Purpose:**

High-energy injuries to the knee may lead to extensive soft tissue loss, fractures, and potential loss of extensor function. The gastrocnemius flap is a prominent reconstructive option for patients with injuries involving the knee and proximal third of the lower extremity. To the best of our knowledge, there has not been an informative review that has evaluated outcomes of patients who have undergone post-traumatic knee reconstruction with a pedicled medial or lateral gastrocnemius flap. The goal of this study is to assess outcomes in patients who have undergone gastrocnemius flap reconstruction after traumatic injuries to the knee.

**Methods:**

The review was conducted using the Preferred Reporting Items for Systematic Reviews and Meta-Analysis (PRISMA) methodology. Four databases were utilized including PubMed, Cochrane Reviews, Embase, and CINAHL. Our search criteria consisted of the following keywords: gastrocnemius, flap, knee, and traum*.

**Results:**

A total of 204 studies were imported for screening, from which five papers met our final inclusion/exclusion criteria. The most common studies utilized in this review were case series followed by retrospective chart reviews. In total, 43 patients with traumatic soft tissue knee defects were included with an average patient age of 27.28 years. All patients had successful and clinical viable flaps post-operatively, and there were a total of five patients who had complications.

**Conclusion:**

The gastrocnemius flap has demonstrated to be an effective option for individuals undergoing post-traumatic knee reconstruction. Infection rates, loss of mobility, and scarring represent a minority of complications that may be seen when this reconstructive technique is utilized. Still, additional randomized controlled trials and retrospective studies are required in order to further evaluate for other potential complications that may occur in this patient population.

## Introduction

Acute knee injuries typically transpire during sudden deceleration, direct impact, and twisting or hyperextension of the knee; those caused by high-energy mechanisms are at higher risk for fractures and vascular injuries [1, 2]. Post-traumatic knee pain is a common complaint that is seen during visits to the emergency department (ED). A study by Gage et al. found that 6,664,324 knee injuries presented to the US EDs from 1999 through 2008, calculated to be an injury rate of 2.29 knee injuries per 1000 individuals [3]. Data from the National Health Fund data between the years 2016 and 2019 demonstrated that the most common traumatic knee injuries were contusion of knee, sprain and tear of other and unspecified parts of knee, and other internal derangements of knee [4].

Traumatic knee injuries are associated with open fractures or dislocations, infections, neurovascular damage, instability, and premature osteoarthritis [5, 6]. Over half of all knee dislocations are due to motor vehicle collisions, but sports injuries and falls from height are other frequent mechanisms of injury [2, 7]. The previous literature has found that the incidence rate of multi-ligamentous knee injuries ranges from 0.001 to 0.013% of all patients assessed for orthopedic injuries [8]. Furthermore, multi-ligament knee injuries and poly-trauma commonly occur in conjunction with one another [9]. The appropriate treatment for traumatic knee injury depends on wound dimensions and geometry, signs of contamination or infection, and the presence of bone, tendon, or implant exposure [10].

Often, orthopedic surgeons face obstacles when addressing soft tissue defect coverage [6]. The introduction of flaps in plastic and orthopedic surgery has largely improved the prognosis of lower extremity defects. Rotational muscle flaps can be used to successfully cover the upper one-third of the tibia, while distant flaps are effective options for complex lower limb wounds [10]. Introduced in 1978, gastrocnemius flaps are a favorable strategy to treat substantial soft tissue defects and have been considered workforce flaps for knee reconstruction due to a reliable axial blood supply and easy of dissection [10–15]. The flap has been utilized for defects secondary to tumor resections, soft tissue complications from total knee arthroplasty (TKA), and post-traumatic injuries [6, 10, 16].

Unlike with TKAs and tumor resections, post-traumatic wounds tend to have additional unforeseen injuries. A study by Kim and Leopold found that surface energy does not necessarily reflect the potential for deep tissue damage, viability, and necrosis [17]. As a result, it is imperative that patients undergo serial wound debridements in order to remove non-viable tissue that may serve as a source of infection [18]. In addition, studies have found that there is a 2%–88% rate of periarticular knee surgical site infections in patients who have suffered periarticular knee fractures [19]. On the other hand, a study by Gausden et al. found that the rate of periprosthetic joint infection (PJI) to be low in patients who suffered from acute wound dehiscence after TKA [20]. Furthermore, these studies demonstrate an increased rate of potential complications that are possible in patients with trauma to the knee in comparison with that of other etiologies requiring knee reconstruction. All in all, trauma to the knee may serve as an obstacle to prompt and successful soft tissue reconstruction.

To the best of our knowledge, there has not been an informative review that has evaluated outcomes of patients who have undergone post-traumatic knee reconstruction with a pedicled medial or lateral gastrocnemius flap. The purpose of this systematic review is to assess the outcomes of patients who have undergone post-traumatic knee reconstruction with a pedicled medial or lateral gastrocnemius flap.

## Methods

The Preferred Reporting Items for Systematic Reviews and Meta-Analyses (PRISMA) guideline was followed throughout the design, implementation, analysis, and reporting of this systematic review, and was registered with the PROSPERO database system [21].

### Search strategies

A search strategy with keyword search terms was built to identify articles pertaining to utilization of the gastrocnemius flap and post-traumatic knee reconstruction. Our search strategy that was applied was: “" gastrocnemius" AND "flap" AND "knee" AND "traum*".” The online databases utilized include PubMed, COCHRANE, EMBASE, and CINAHL. There were no restrictions when conducting the search regarding publication date, study language, or study type.

### Study selection

The identified articles were then imported into the COVIDENCE software, an online application tool used for primary screening and data extraction. Once all duplicates were removed, studies were selected based on our inclusion/exclusion criteria: English-language only, no systematic reviews, no case reports, and published literature that focused solely on post-traumatic knee reconstruction. Two independent reviewers (RG and JW) selected the studies through title and abstract screening. All conflicts were resolved by a third-party individual (EL). Once irrelevant studies had been removed from the study group, the papers underwent full-text review by two independent reviewers (RG and JW), with a third reviewer resolving any conflicts (EL).

### Quality assessment

The methodological index for non-randomized studies (MINORS) guidelines was used to assess the quality of selected manuscripts. The MINORS guidelines are composed of 12 questions including a clear stated aim, inclusion of consecutive patients, prospective collection of data, endpoints appropriate to the aim of the study, unbiased assessment of the study endpoint, follow-up period appropriate to the aim of the study, loss to follow-up less than 5%, prospective calculation of the study size, an adequate control group, contemporary groups, baseline equivalence of groups, and adequate statistical analyses [22]. Each item has a value of up to 2, with the ideal score being 16 for non-comparative studies and 24 for comparative studies. Three independent reviewers (RG, JW, and EL) scored each study, and scores were averaged between the reviewers and rounded to the closest whole number.

### Data extraction

Two review authors (RG and JW) independently extracted data from eligible studies using a standardized extraction form which recorded the following information:

1. Methods: study type, level of evidence, and sample size
2. Participants: year of study, sample size, age of participants, gender, and procedural indication
3. Intervention: the technique used, details of the procedure, and follow-up period
4. Outcomes: post-procedural complications, reoperations, esthetic outcome, patient satisfaction, and long-term complications

### Data analysis

Once all of the data had been extracted, it was then imported into Microsoft Excel (Microsoft Office Excel 2022 Redmon, WA, USA). Microsoft Excel was then utilized to compile data, and create tables for the manuscript.

## Results

### General overview

**Fig. 1:**
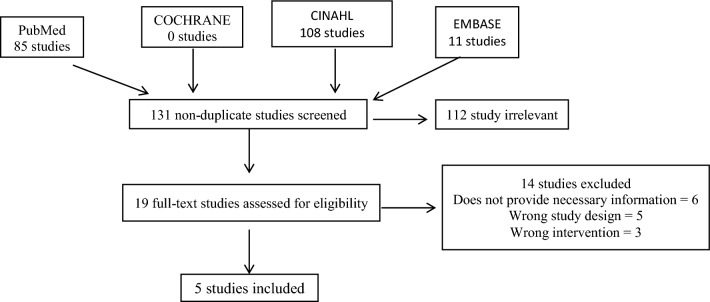
Flowchart illustrating study selection based upon PRISMA-P guidelines

The study selection PRISMA flow diagram is shown in Fig. 1. A total of 204 studies were imported for screening, from which 73 duplicates were removed. One hundred and thirty-one studies were screened, and 112 studies were screened as irrelevant. Nineteen full-text studies were assessed, and five papers met our final inclusion/exclusion criteria (Fig. 1). Quality assessment was performed on the selected studies (Table 1). There was a lack of control groups in the studies that were included in this manuscript, preventing a meta-analysis to be performed.

**Table 1:** Design, characteristics, and quality (using MINORS score) of included gastrocnemius flap studies

Study	Study design	Level of evidence*	Indications for surgery	MINORS score	Statistical analysis
Saaiq and Zimri (2019)^21^	Case series	IV	Traumatic defect of the knee	7	No
Gkiatas et al. (2021)^6^	Retrospective chart review	III	Traumatic defect of the knee	13	No
Hohmann et al. (2016)^22^	Retrospective chart review	III	Traumatic defect of the knee	13	No
Leung et al. (1994)^23^	Case series	IV	Traumatic defect of the knee	6	No
Asko-Seljavaara and Haajanen (1982)^24^	Case series	IV	Traumatic defect of the knee	8	No

*Oxford Center for Evidence-Based Medicine—level of evidence for the included studies

### Clinical characteristics analysis

In total, five studies were utilized for the clinical characteristics analysis, three of which were case series, and two of which were retrospective chart reviews (Table 2). The included studies discussed a similar surgical technique. Between the five studies, the number of patients varied, ranging from 4 to 20. The range of patient age was vast, spanning from 5 to 79 years. Flap survival was 100% in all studies, and there were only two studies (Hohmann et al. and Asko-Seljavaara and Haajanen) that reported patient complications [23, 24]. Studies by Saaiq and Zimri and Leung et al. failed to mention information in regard to post-operative follow-up [25, 26]. For the remaining studies, the follow-up ranged from 6 months to 5.1 years on average [6].

**Table 2:** Overview of included gastrocnemius flap studies

Study	Study period	Number of patients	Age at intervention	Flap survival rate (%)	Overall incidence of complications	Follow-up	Conclusion
Saaiq and Zimri (2019)^21^	2015–2018	20	16–53	100	Not mentioned	Not mentioned	Gastrocnemius muscle flap was a quick, easy, and reliable coverage too for small to moderate traumatic knee defects
Gkiatas et al. (2021)^6^	Not mentioned	10	17–79	100	None	4.4 years	Gastrocnemius muscle transfer is a useful technique for coverage of traumatic soft tissue defects involving the knee and upper tibia
Hohmann et al. (2016)^22^	Not mentioned	4	28–40	100	100%	5.1 years	The medial gastrocnemius flap is an excellent reconstructive option for severe traumatic knee injuries with soft tissue defects and extensor mechanism disruptions
Leung et al. (1994)^23^	Not mentioned	4	28–60	100	Not mentioned	Not mentioned	The gastrocnemius flap is an effective treatment option in order to enhance wound healing with minimal complications in patients with traumatic knee defects
Asko-Seljavaara and Haajanen (1982)^24^	Not mentioned	5	5–53	100	20%	0.5 years	The gastrocnemius flap is a reliable option in both emergent and chronically infected cases with exposed knee joint cavities

The aggregate data determined that there were a total of 43 patients with an average age of 27.28 years (Table 3). Twenty-four patients failed to have any mention of complications while there were 14 patients who had no complications. Five patients had complications with one patient having knee effusion for 2 months post-operatively, two patients with infection, two patients with impaired knee range of motion, one patient with a prevalent post-surgical scar, and one patient who had bony malalignment secondary to injuries to the lower extremity.

**Table 3:** Age of study participants and type of complications observed in included studies

N	43
Average age	27.28
*Overview of complications*	
Not discussed	24
Yes	5
None	14
*Observed complications*	
Knee effusion	1
Infection	2
Impaired range of motion	2
Prevalent scar	1
Bony malalignment	1

One study by Gkiatas et al. mentioned the mean covered area of deficits to be 62.4 cm^2^ with the range being from 36 to 144 cm [2, 6]. In addition, the authors report that all patients received split-thickness skin grafts (STSG) at the time of the initial procedure with no complications. Lastly, all patients had satisfactory coverage of defects, and all were pleased with their surgical outcome. Another study by Hohmann et al. discussed knee range of motion after surgical intervention and found that range of motion ranged from 0 to 120° [23]. They determined that all except one patient was able to return to physical activity due to severity of the injury. All in all, Hohmann et al. determined that the pedicled gastrocnemius flap was successfully able to reprise the role as an extensor in the lower extremity.

## Discussion

We conducted a systematic review of reported literature on patients who underwent gastrocnemius flap for reconstruction for post-traumatic knee injuries. To the best of our knowledge, there has not been a published systematic review that has evaluated the outcomes of patients who have undergone post-traumatic knee reconstruction with a pedicled medial or lateral gastrocnemius flap. This is a topic of great significance, as the pedicled gastrocnemius flap is able to mitigate the need for free tissue transfers and above-knee amputations (AKA) in patients with severe trauma to the knee. Furthermore, the limited availability of randomized controlled trials and retrospective studies makes it difficult to assign this systematic review as the highest quality of evidence.

The management of soft tissue defects in the knee is a challenging topic [27]. More importantly, high-energy trauma to the knee typically results in substantial soft tissue defects along with Gustilo–Anderson IIIB or IIIC open fractures [28]. In addition, the knee is a crucial weight-bearing and mobile joint in the lower extremity, requiring peri-knee soft tissue reconstruction to have adequate flexibility and durability [29]. In our study, we found that utilization of the gastrocnemius flap for post-traumatic knee reconstruction led to adequate range of motion in 75% of patients. Hohmann et al. determined that most patients undergoing post-traumatic knee reconstruction with the gastrocnemius flap were successfully able to ambulate after the procedure [23]. However, they found that in patients with more severe injuries, there was limited mobility regardless of rehabilitation and other conservative measures that were undertaken [23]. Other studies that have utilized the gastrocnemius flap for other etiologies requiring knee reconstruction and have noted similar renewed mobility when the gastrocnemius flap has been used [30, 31]. A study by Zhang et al. determined that patients with severe damage to the articular surfaces of the patella were often limited in their range of motion of the knee regardless of wound etiology [30]. In patients with traumatic injuries to the knee, patellar damage is likely, although it was not specifically reviewed in the studies that were included in this systematic review.

Wound debridement and irrigation has been referred to as one of the pillars in the management of soft tissue defects in order to reduce the rates of infection [32]. In an included study by Hohmann et al., the authors report that all patients underwent irrigation and debridement prior to definitive reconstruction, which occurred at an average of 32 days after initial injury. The reported number of debridements varied, ranging from 2 to 5 [23]. Saaiq and Zimri reported that along with irrigation and debridement, they also utilized vacuum-assisted closure in wounds that were contaminated prior to proceeding with surgical management [25]. Our report demonstrated that only 2 patients (10.5%) suffered from post-operative infections related to the surgical procedure, illustrating the importance of irrigation and debridement prior to reconstruction in patients with traumatic knee injuries. Other studies have found similar findings including Flood et al. who determined that arthroscopic irrigation and debridement successfully led to no clinical or radiographic evidence of infection and knee function and range of motion returning [33].

Approximately 26.3% of patients in this study had complications related to the surgical procedure including impaired range of motion, infection, prominent effusions, and scarring. This rate of complications is significantly lower than what has been reported in the literature (59.9%) when the gastrocnemius flap has been used for other indications such as for total knee arthroplasty (TKA) [34]. Additionally, the observed rate of infection (10.5%) in this study is lower than that of what has been reported in the literature [35, 36]. It is important to take into consideration that higher rates of post-operative infection may be due to high-energy injuries that lead to significant vascular damage and wound contamination [37]. Two patients had decreased range of motion, which was attributed to the severity of the injury rather than that of the flap transfer. This has been shown in a study by Diageler et al. who found that in their study, functional impairment and strength loss in patients was the result of infection, preceding trauma, or resection of tumors [16]. Furthermore, the flap survival rate in this study was 100%, which is similar to other reported results when the gastrocnemius flap has been utilized for other uses such as in TKAs [38]. This further demonstrates the effectiveness and feasibility of successfully utilizing the gastrocnemius flap in patients who may require various degrees of soft tissue reconstruction due to traumatic knee injuries.

There are several limitations of this review that must be taken into account. Primarily, it is difficult to evaluate for patient satisfaction, complications, and surgical results in a few of these studies. All the included manuscripts failed to identify patient satisfaction post-operatively with well-established patient satisfaction scoring systems. There was one manuscript by Gkiatas et al. who discussed that all patients were pleased with their surgical result but failed to utilize a formalized scale [6]. In order to combat this issue, quality assessment methods should be standardized to have a uniformed reporting system. In addition, it is important to take into account that surgical bias exists and has the potential to be a factor that cannot be excluded from this study. Also, our literature review failed to include prospective studies, as there was limited published literature regarding utilization of the gastrocnemius flap for post-traumatic knee reconstruction. The majority of studies were case series, and there were no randomized controlled trials, which made it difficult to compare the efficacy of the respective treatment approach. Furthermore, across the studies included, there was not a uniform way in which data were presented causing inconsistencies in reported information across studies. The inclusion of only English-language publications in the literature search presents an important potential source of bias as valuable data and insights from non-English publications are precluded which may affect the generalizability of the review's conclusions. Additionally, the overall quality of the included studies for this review warrants consideration. While efforts were made to assess the methodological quality of the studies using the MINORS guidelines, it is important to acknowledge that the included studies varied in design and reporting standards. This variability could impact the reliability and validity of the data extracted for analysis, potentially influencing the robustness of the review's conclusions. In order to address these various issues, it is critical for studies to be both larger and more standardized in order to further various other findings within this topic.

## Conclusion

The gastrocnemius flap has become a workhorse for reconstruction of the knee and defects involving the proximal third of the tibia. However, there are limited studies that have studied the effects of this reconstruction method on patients who have suffered traumatic knee injuries with substantial soft tissue defects. The gastrocnemius flap has demonstrated to be a successful, safe, and effective reconstructive option in patients with traumatic knee injuries. The rate of complications is minimal, flap success rate was found to be 100%, and post-surgical knee range of motion has demonstrated to be renewed. Still, prospective studies and randomized control trials are needed to further define the efficacy of this procedure.
